# Patiromer for Patients With Heart Failure at Risk of Hyperkalemia: CARE-HK in HF Registry

**DOI:** 10.1016/j.cardfail.2026.03.025

**Published:** 2026-03-26

**Authors:** OMAR CANTU-MARTINEZ, STEPHEN J. GREENE, KHAJA M. CHINNAKONDEPALLI, STEFAN D. ANKER, BIYKEM BOZKURT, JAVED BUTLER, JOHN G. CLELAND, NIHAR DESAI, DIEDERICK E. GROBBEE, ELLIE KELEPOURIS, GIUSEPPE ROSANO, TIBOR KEMPF, MARTIN SCHULZ, CARLA-MARIA BRANDES, VICTORIA DONACHIE, ANDREW J. SAUER

**Affiliations:** 1 Saint Luke’s Mid America Heart Institute and University of Missouri, Kansas City, Missouri; 2 Duke Clinical Research Institute, Durham, North Carolina; 3 Division of Cardiology, Duke University School of Medicine, Durham, North Carolina; 4 Department of Cardiology (CVK) of German Heart Center Charité, German Centre for Cardiovascular Research (DZHK) partner site Berlin, Charité, Universitätsmedizin, Berlin, Germany; 5 Winters Center for Heart Failure Research, Cardiovascular Research Institute, Baylor College of Medicine, Houston, Texas; 6 Baylor Scott and White Research Institute, Dallas, Texas; 7 University of Mississippi, Jackson, Mississippi; 8 School of Cardiovascular and Metabolic Health, University of Glasgow, Glasgow, UK; 9 Section of Cardiovascular Medicine, Yale School of Medicine, Center for Outcomes Research and Evaluation, Yale New Haven Hospital, New Haven, Connecticut; 10 Julius Global Health, Julius Center for Health Sciences and Primary Care, University Medical Center, Utrecht University, Utrecht, the Netherlands; 11 Division of Renal Electrolyte and Hypertension, University of Pennsylvania, Philadelphia, Pennsylvania; 12 Department of Human Sciences and Promotion of Quality of Life, San Raffaele Open University of Rome, Rome, Italy; 13 IRCCS, San Raffaele Roma, Italy; 14 Department of Cardiology and Intensive Care Medicine, Städtisches Klinikum Braunschweig, Braunschweig, Germany; 15 Department of Medicine, ABDA – Federal Union of German Associations of Pharmacists, and Institute of Pharmacy, Freie Universität Berlin, Berlin, Germany; 16 CSL Vifor, Glattbrugg, Switzerland.

**Keywords:** Hyperkalemia, patiromer, heart failure, RASi optimization, serum potassium, chronic kidney disease

## Abstract

**Background:**

For patients with heart failure (HF), hyperkalemia (HK) may prevent optimal renin–angiotensin system inhibitor (RASi) use. Randomized trials show that patiromer, a contemporary potassium binder, reduces serum potassium (sK^+^) concentrations and HK, potentially enabling RASi optimization. We sought to assess the real-world effectiveness of patiromer in facilitating RASi optimization in HF patients at increased risk of HK.

**Methods and Results:**

The CARE-HK in HF (Cardiovascular and Renal Treatment in HF Patients with HK or at High Risk of HK) (NCT04864795) was a prospective registry of patients with chronic HF at increased risk of HK (prior or current HK or estimated glomerular filtration rate of <45 mL/min/1.73 m^2^). Patients treated with patiromer were propensity score matched with others not receiving potassium binders. HK episodes, sK^+^, and RASi optimization (≥50% target doses) were evaluated prospectively and retrospectively. Among 2558 patients, 234 (9.1%) received patiromer for a median of 12.0 months (interquartile range 6.0–19.4 months). Propensity matching yielded 211 patients treated with patiromer and 361 matched controls. The median patient age was 73 years (interquartile range 64–79 years); 79.4% were men, 50.2% had diabetes, and 44.9% had an estimated glomerular filtration rate of <45 mL/min/1.73 m^2^. At patiromer initiation, the median sK^+^ was 5.5 mEq/L (interquartile range 5.4–5.7 mEq/L) and declined by 0.21 mEq/L (95% confidence interval 0.30–0.12 mEq/L) (*P* < .0001). However, RASi/mineralocorticoid receptor antagonist optimization within 2 years remained at ≤40% and did not increase with patiromer.

**Conclusions:**

In contemporary clinical practice, patiromer was associated with reducing sK^+^ but rates of RASi/mineralocorticoid receptor antagonist optimization remain low. Overcoming clinical inertia, including the addition of patiromer when appropriate, is required to optimize RASi/mineralocorticoid receptor antagonist therapy in patients with HF at high risk of HK.

## Lay Summary

- Many patients with heart failure remain undertreated with renin–angiotensin–aldosterone system inhibitors owing to concerns over hyperkalemia.
- Patiromer effectively lowers serum potassium concentration, particularly in patients with moderate to severe hyperkalemia.
- Overcoming therapeutic inertia remains problematic despite the availability of effective agents to prevent or treat hyperkalemia.

Real-world evidence consistently shows that patients with heart failure (HF) remain undertreated, with many not achieving target doses of guideline-directed medical therapy (GDMT).^1–4^ Side effects of renin–angiotensin system inhibitors (RASis) and mineralocorticoid receptor antagonists (MRAs), including the risk of hyperkalemia (HK), contribute to this treatment gap.^1,3,5–8^ Serum potassium (sK^+^), has a U-shaped relationship with mortality with a nadir of risk at around 4.5 mEq/L.^9–11^ Maintaining sK^+^ in the optimal range and optimal implementation of GDMT in those at risk of HK should be associated with better outcomes.^12–14^

Contemporary potassium binders, such as patiromer and sodium zirconium cyclosilicate, enable patients with HF at high risk of HK to achieve and maintain optimal GDMT.^6^ PEARL-HF (Parallel Evaluation of RLY5016 in Heart Failure) showed that, in patients with HF who had discontinued RASi subsequent to an episode of HK, patiromer reduced sK^+^ and the rate of HK, and increase the proportion of patients tolerating optimal MRA.^15^ DIAMOND (Patiromer for the Management of Hyperkalemia in Subjects Receiving RAASi for HFrEF) demonstrated that patiromer helped to achieve optimal doses of RASi/MRA therapy and reduced HK episodes.^16^

Despite the introduction of newer, better-tolerated HF therapies that decrease the risk of HK,^17,18^ its recurrence remains high, leading to increased hospitalization rates and costs.^6,19^ Although randomized trials have demonstrated the benefits of potassium binders, the extent to which these findings translate into effectiveness in clinical practice remains unclear. The CARE-HK in HF (Cardiovascular and Renal Treatment in HF Patients with HK or at High Risk of HK) registry (NCT04864795) enrolled patients at risk for HK to evaluate the effectiveness of patiromer and its association with RASi/MRA treatment patterns.^20^ By mitigating HK risk, contemporary potassium binders could help to optimize the use of RASi/MRA in patients with HF, thereby improving clinical outcomes.^21^

## METHODS

### Study Design and Population

CARE-HK in HF was a prospective, multinational, noninterventional registry of outpatients with HF (regardless of left ventricular ejection fraction) treated with a RASi and treated with or eligible for MRA and at increased risk of HK, defined as current or prior HK or an estimated glomerular filtration rate of <45 mL/min/1.73 m^2^.^20^ Patients were enrolled between April 6, 2021, and August 31, 2023. RASi use including any of angiotensin-converting enzyme inhibitors (ACEis), angiotensin II receptor blockers (ARBs), angiotensin II receptor-neprilysin inhibitors (ARNis,) and MRA use and optimization was assessed. An increased risk of HK was defined as ≥1 of the following: sK^+^ >5.0 mEq/L at enrollment, a history of HK >5.0 mEq/L within the prior 24 months, and/or an estimated glomerular filtration rate of <45 mL/min/1.73 m^2^, that is, chronic kidney disease (CKD) stage ≥3b. HK was defined as an sK^+^ concentration of >5.0 mEq/L with mild, moderate, and severe HK defined as sK^+^ values of >5.0 to ≤5.5, >5.5 to ≤6.0, and >6.0 mEq/L, respectively. Optimal RASi/MRA was defined as reaching ≥50% of the guideline-recommended dose for ≥1 ACEi/ARB/ARNi, and MRA. Suboptimal RASi/MRA was defined as having <50% of the guideline-recommended dose for all ACEi/ARB/ARNi, or MRA, including untreated patients. The registry period included a retrospective component, in which data on RASi/MRA use, dosing, and episodes of HK were collected for ≤24 months before enrollment, and a prospective follow-up period of ≥6 months after enrollment. Patients’ baseline characteristics, RASi/MRA modifications after an HK episode, sK^+^ values, and other relevant data were collected from medical records and recorded in an electronic case report form. Participating sites did not require study-specific protocols, and all medical decisions were at the discretion of the treating clinicians.^20^ For this analysis, patients were stratified and propensity score matched by patiromer use, as patiromer treated and not-treated with a potassium binder. Patients classified as not treated with potassium binder, that is, the control group, did not receive any potassium binder during their participation in this registry. The protocol received approval from the institutional review boards or ethics committees at all participating sites, and written informed consent was obtained from all participants.

### Sample Size Calculation

A 10% improvement in RASi/MRA treatment optimization with patiromer was deemed clinically meaningful. The sample size calculation, using a 2-sample test for independent proportions, assumed an optimization rate of 45% in the control group, with an improvement to 55% in the patiromer-treated group. Under these assumptions and using a 1:2 ratio of patiromer-treated to patients not treated with potassium binders, approximately 300 patiromer-treated patients were required to achieve 80% power at a 5% significance level.

### Propensity Score Matching

Patients treated with patiromer were propensity score matched with patients who had ≥1 HK episode and did not receive potassium binder therapy. Patients were excluded from the not treated with potassium binder group for this analysis if no HK episode was reported after RASi/MRA initiation. Although the study design anticipated approximately 300 patiromer-treated patients for 1:2 matching, early termination of the CARE-HK in HF registry resulted in fewer patiromer-treated patients being enrolled and an overall low use of potassium binders.

Propensity scores were estimated using the SAS PSMATCH procedure with a multivariable logistic regression model, in which receipt of patiromer was the dependent variable and prespecified baseline covariates were included as independent variables. A single propensity score was estimated for the entire study population and used consistently across all matched analyses. In cases where model convergence issues occurred, covariates with sparse categories were collapsed; if convergence issues persisted, covariates contributing to nonconvergence were removed.

Matching was performed using optimal variable-ratio matching targeting a 1:2 treated-to-control ratio (1 patiromer-treated patient to ≤2 control patients). Closeness between treated and control patients was defined by the absolute difference in the logit of the propensity score, and a caliper width of 0.25 times the pooled standard deviation of the logit of the propensity score was applied. When <2 eligible control matches were available within the predefined caliper, treated patients were matched with a single control rather than excluded.

The covariates considered for propensity score matching included demographics, reason for RASi/MRA at enrollment, time since RASi/MRA initiation, comorbidities, sK^+^ concentration, other laboratory data, left ventricular ejection fraction percent, New York Heart Association functional class, and available follow-up time. For a complete list of covariate balance assessments, including standardized mean differences and variance ratios from the propensity score model, refer to Supplementary Table S1.

Laboratory data values, including sK^+^ concentrations, were captured on the analysis date. For the patiromer-treated group, the analysis date was defined as the time of the last HK episode before or at the time of patiromer initiation. For the control group, the analysis date was defined as the time of the first HK episode reported during the registry period. Follow-up time for patiromer-treated patients was calculated as the difference between the follow-up end date and the date of patiromer initiation. Follow-up time for patients not treated with a potassium binder was calculated as the difference between the follow-up end date and the first HK episode date after RASi/MRA initiation for HF.

### Statistical Analyses

Patiromer and other potassium binders use, and the reasons for use were reported as frequencies and percentages. Potassium binder frequencies in the United States and Europe were compared using chi-square tests or exact tests, as appropriate. The frequencies and percentages of any changes to assess RASi/MRA modifications after an HK episode in patiromer-treated patients before patiromer initiation were described. All patiromer-treated and not-treated with potassium binder patients’ baseline characteristics were described, with continuous variables reported as median (25th–75th percentile) and categorical variables reported as frequencies and percentages. To assess the change in sK^+^ before and after patiromer initiation, estimated by the slope difference in mEq/L/month in the patiromer-treated cohort, a piecewise linear mixed model was performed with sK^+^ values as the dependent variable, time (continuous, in months, calculated as the difference between sK^+^ measurement date and patiromer initiation date divided by 30.4), and a time spline (equal to 0 before patiromer initiation, and equal to time after patiromer initiation) as fixed effects, with patient as a random effect. For this linear mixed-effects analysis, sK^+^ measurements were restricted to values obtained within 3 months before and ≤6 months after patiromer initiation to capture clinically relevant pretreatment values and post-treatment response. For analyses by subgroups of sK^+^ ranges, the subgroup variable (categorical) and its interaction with time and time spline were added as fixed effects, with the patient’s patiromer initiation date used as a knot. To compare the proportion of matched patients achieving RASi/MRA optimization over time (6-month intervals) in patiromer-treated vs matched patients not treated with a potassium binder, rates of optimal and suboptimal RASi/MRA therapy were summarized as frequencies and percentages in both groups. Inferential comparisons were performed using conditional logistic regression, conditioning on the matched sets at each time interval to account for the dependence caused by propensity score matching.

To assess the association between patiromer use and RASi/MRA optimization after an HK episode, a mixed-effects logistic regression analysis was performed to estimate the probability of suboptimal RASi/MRA treatment (which includes those not treated) vs optimal RASi/MRA treatment. Treatment group was included as a fixed effect, and random intercepts for patient and matched set were included to account for within-subject correlation owing to repeated measures and dependence induced by propensity score matching, respectively. The model included a covariate for time (6-month time intervals), with the not-treated with potassium binders group serving as the reference. Any variable for which balance was not achieved after propensity score matching was added as a covariate, with covariates assessed at the time of the analysis date (as defined in the propensity score matching section). Changes in treatment effect across time intervals were explored by including treatment-by-visit interaction terms. For analyses of RASi/MRA optimization, optimal, suboptimal/no-treatment status was defined according to the level of optimization category achieved for the majority of each 6-month period.

### Sensitivity Analysis

To account for additional interventions for HK, a sensitivity analysis was conducted by adding patients treated with sodium zirconium cyclosilicate to those treated with patiromer in the piecewise linear mixed model previously limited to patiromer. Their sK^+^ slope differences were evaluated across baseline sK^+^ strata in the patiromer and sodium zirconium cyclosilicate-treated patients (unmatched population). Owing to infrequent use, patients treated with calcium and sodium polystyrene sulfonate were not added to this analysis of patients treated with either patiromer or sodium zirconium cyclosilicate.

A two-tailed *P* value of <.05 is generally considered statistically significant; however, this was an exploratory analysis, and reported *P* values should be interpreted in this context and with caution. Statistical analyses were done using SAS version 9.4 (SAS Institute, Cary, NC).

## RESULTS

### Real-world Use of Patiromer and Other Potassium Binders

Among 2558 total patients in CARE-HK across 8 countries, 494 uses of potassium binders were reported in 434 patients (17.0%). Of the total patients, 234 (9.1%) received patiromer for a median total duration of 12.0 months (interquartile range 6.0–19.4 months). Other potassium binders used among enrolled patients included sodium zirconium cyclosilicate, prescribed in 150 (5.9%), calcium polystyrene sulfonate, prescribed in 62 (2.4%), and sodium polystyrene sulfonate, prescribed in 48 (1.9%) patients. For all four agents, the most common reason for use was to treat HK (85%, 79.3%, 88.7%, and 93.8%, respectively), while use for purposes of maintaining normokalemia, titrating, or initiating RASi/MRA was less common (Supplementary Table S2).

Patient characteristics of patiromer-treated patients and those not treated with potassium binders (control group) before propensity score matching are shown in Supplementary Table S3. Most patients who received patiromer (*n* = 234) were enrolled at European sites (*n* = 212 [91%]), with fewer patients enrolled at US sites (*n* = 22 [9%]), consistent with the geographic distribution of the study cohort. Additionally, among RASi-treated patients with a recent HK episode included in Supplementary Table S4 (*n* = 72), there were no RASi/MRA modifications within 3–30 days after the episode in 94.4% of cases before patiromer initiation.

### Propensity Score Matching and Patient Characteristics of Patiromer-treated Patients and Patients Not Treated With a Potassium Binder

**Fig 1.**
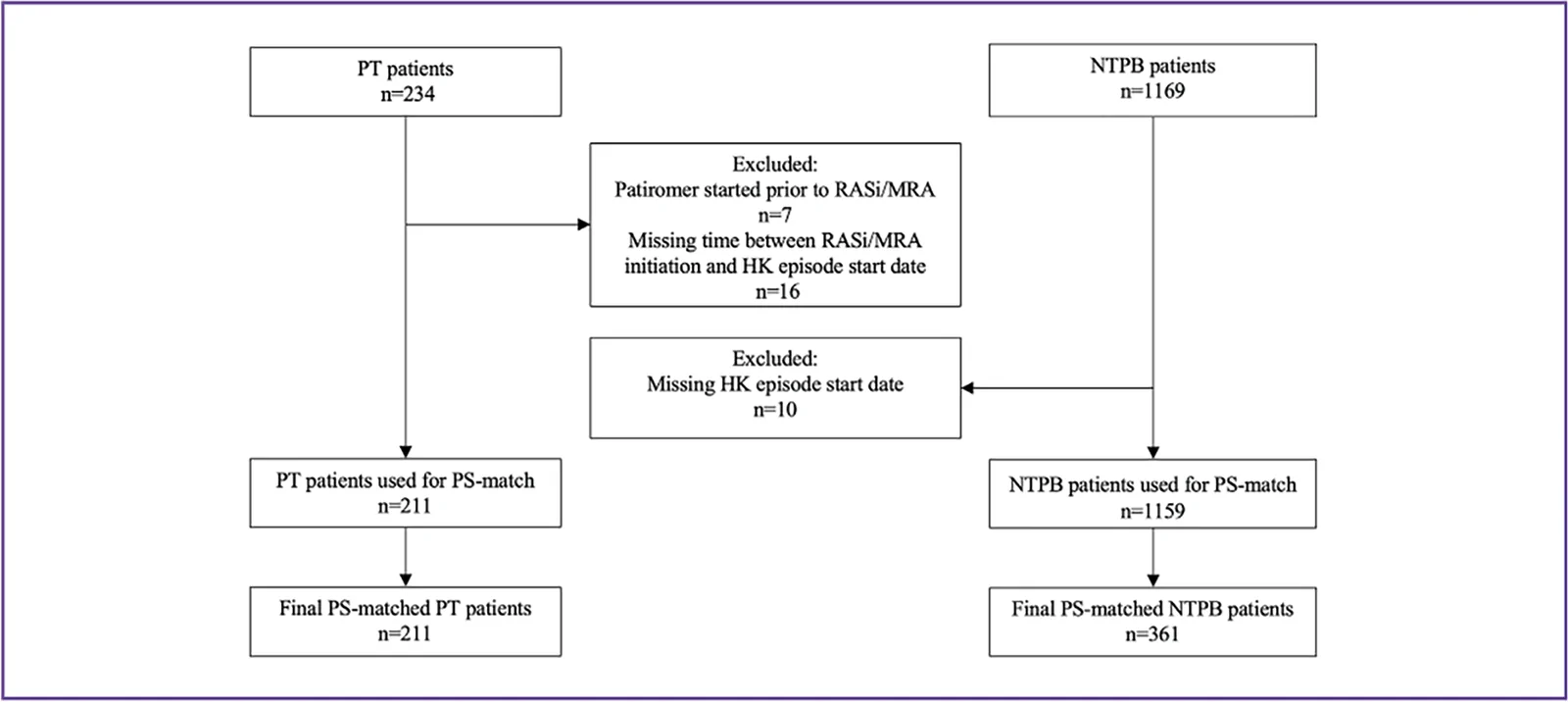
Flowchart for matching patiromer-treated patients and those not treated with a potassium binder. HK, hyperkalemia; MRA, mineralocorticoid receptor antagonist; NTPB, not treated with potassium binder; PS, propensity score; PT, patiromer treated; RASi, renin–angiotensin system inhibitor.

Propensity score matching yielded 211 patiromer-treated and 361 control patients not treated with a potassium binder (Fig. 1). Although a 1:2 matching ratio was targeted, not all patiromer-treated patients had two eligible control matches within the prespecified matching criteria, resulting in some treated patients being matched to only one control. The propensity score model, as described in Supplementary Table S1, was successful in achieving good covariate balance for most variables, except for a minor imbalance in log-transformed sK^+^ values at the time of the HK episode (standardized mean difference 0.18), and hence subsequent matched analyses were adjusted for this imbalanced covariate. After propensity score matching, the median total treatment duration of patiromer was 11.4 months. Patiromer-treated patient disposition overall and by region, as well as treatment durations before and after propensity score matching, are shown in Supplementary Table S5.

After propensity score matching, the median age was 73 years (interquartile range 64–79 years); most were male (79.4%), White (88.3%), and had an estimated glomerular filtration rate of <45 mL/min/1.73 m^2^ (44.9%), and with >3 comorbidities (67.0%) (Table 1). The median baseline sK^+^ values were similar between groups (patiromer-treated 5.5 mEq/L; not treated with a potassium binder 5.4 mEq/L). The median follow-up duration was 12.3 months for patiromer-treated patients and 13.8 months for patients not treated with a potassium binder.

### sK^+^ Concentration Changes in All Patiromer-treated Patients

**Fig 2.**
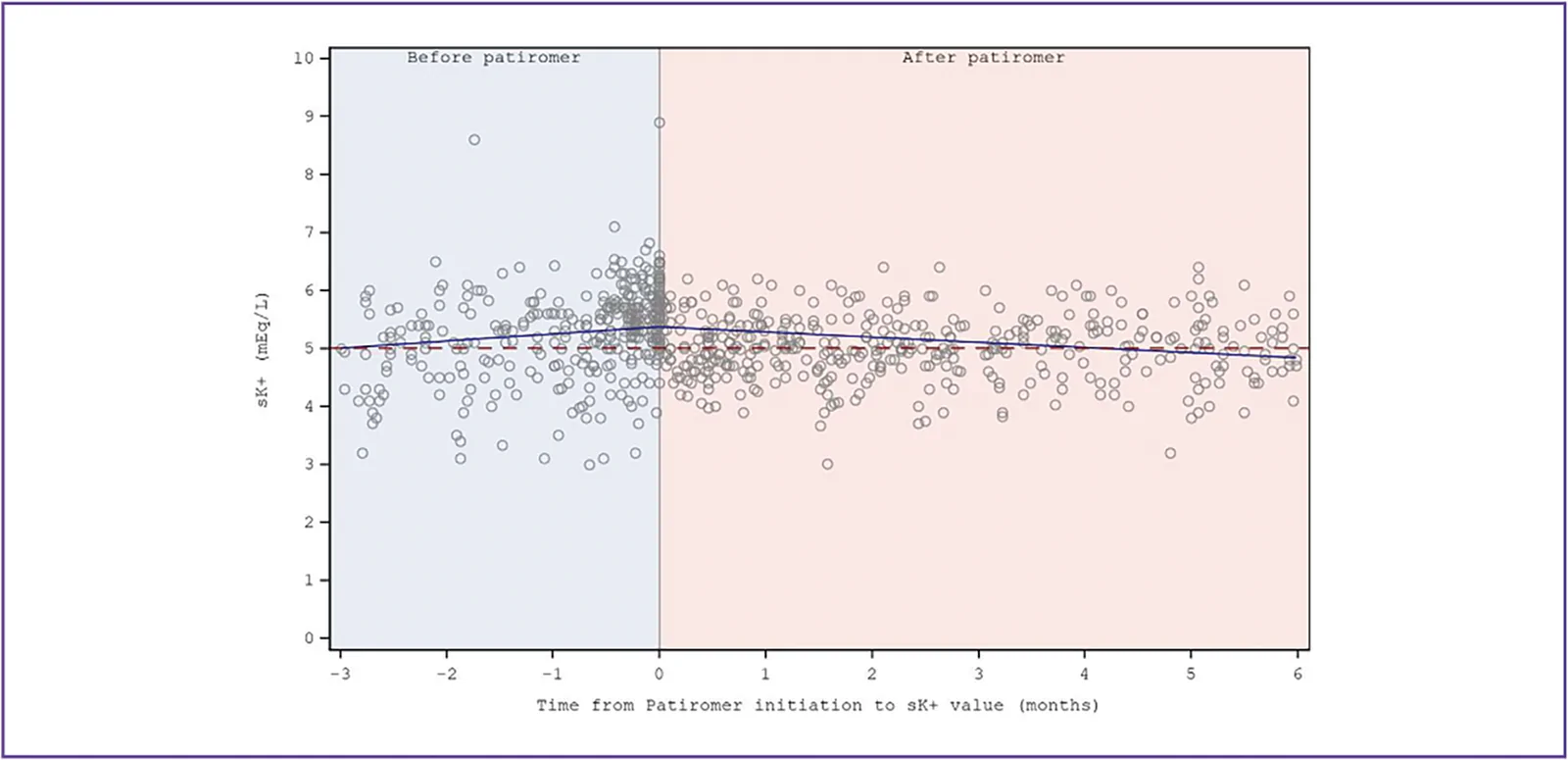
Piecewise linear mixed model of serum potassium values before and after patiromer initiation of all patiromer-treated patients (*n* = 234). Linear mixed model with sK^+^ values as the dependent variable, time (continuous, in months, calculated as the difference between sK^+^ measurement date and patiromer initiation date divided by 30.4), time spline (equal to 0 before patiromer initiation, and equal to time after patiromer initiation) as fixed effects and patient as random effects. Patiromer initiation date is used as a knot. The blue line indicates the regression line. The red dashed line indicates the sK^+^ concentration threshold for hyperkalemia at 5 mEq/L. The circles in the figures illustrate all sK^+^ readings; all results for a patient are included, so there are multiple sK^+^ readings displayed. sK^+^ values measured before 3 months or after 6 months from patiromer initiation were excluded from the analysis. Values below 2 mEq/L and above 9 mEq/L were considered to be clinically invalid and are excluded from the analysis. Duplicated values of sK^+^ reported on the same day for the same patient were excluded from analysis, only distinct values were considered in the models. sK^+^, serum potassium.

**Fig 3.**
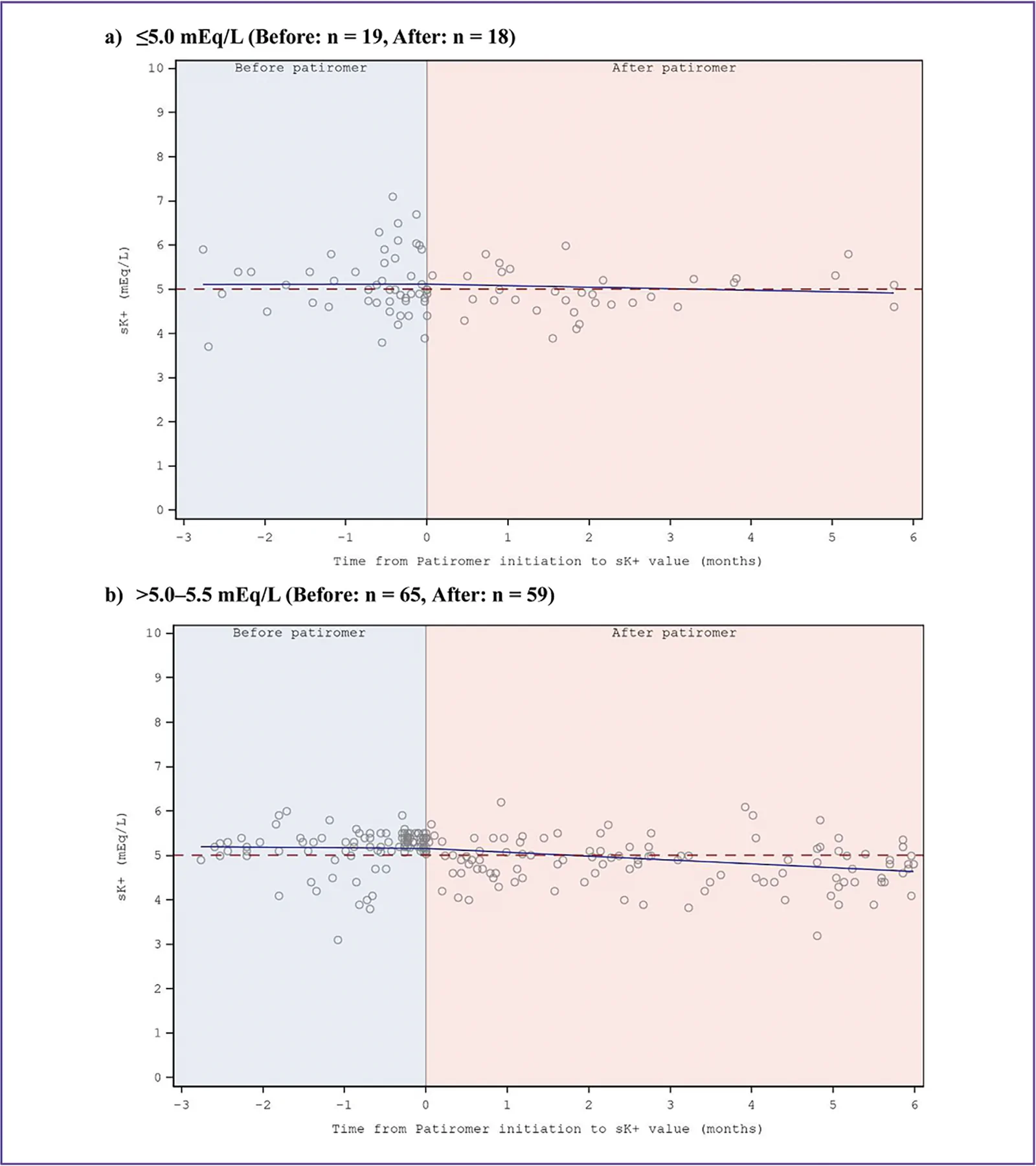

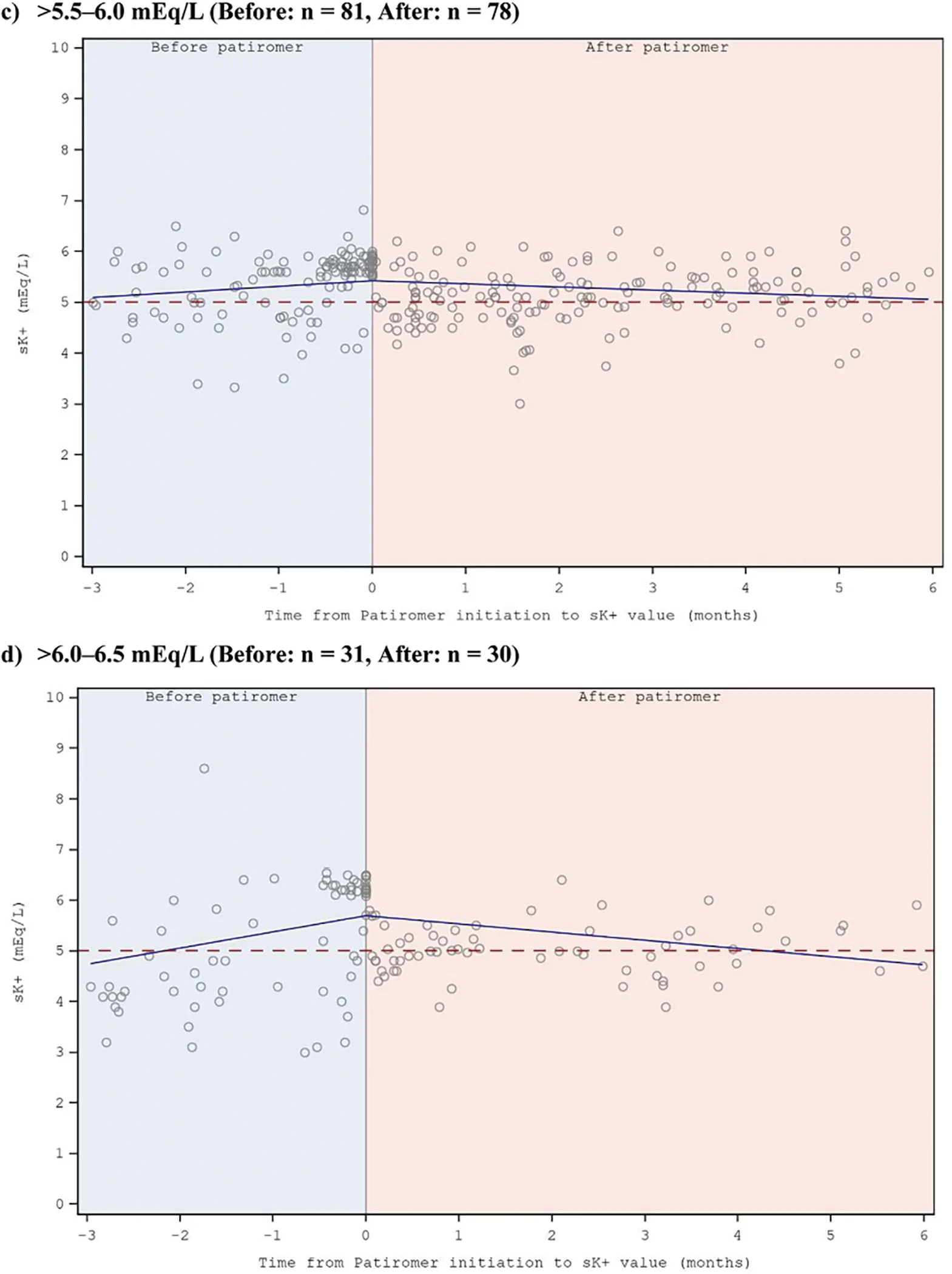
Piecewise linear mixed model of sK^+^ before and after patiromer initiation, with subanalyses by sK^+^ category of all patiromer-treated patients (*n* = 196). Linear mixed model with sK^+^ values as the dependent variable, time (continuous, in months, calculated as the difference between sK^+^ measurement date and patiromer initiation date divided by 30.4), time spline (equal to 0 before patiromer initiation, and equal to time after patiromer initiation) as fixed effects and patient as random effects. sK^+^ subgroup variable (categorical) and its interaction with time and time spline were added as fixed effects. Patiromer initiation date is used as a knot. The blue line indicates the regression line. The red dashed line indicates the sK^+^ concentration threshold for hyperkalemia at 5 mEq/L. The circles in the figures illustrate all sK^+^ readings; all results for a patient are included, so there are multiple sK^+^ readings displayed. sK^+^ values measured before 3 months or after 6 months from patiromer initiation were excluded from the analysis. Values below 2 mEq/L and above 9 mEq/L were considered to be clinically invalid and are excluded from the analysis. Duplicated values of sK^+^ reported on the same day for the same patient were excluded from analysis, only distinct values were considered in the models. sK^+^, serum potassium.

Before propensity score matching, the median sK^+^ at the time of patiromer initiation was 5.6 mEq/L (interquartile range 5.3–5.8 mEq/L) in all patiromer-treated patients (*n* = 234). sK^+^ values before and after patiromer initiation, including missing sK^+^ at each period, are shown in Supplementary Table S6. In the linear mixed model, patiromer initiation was associated with a reduction in sK^+^, with larger reductions in sK^+^ observed when patiromer was initiated at higher sK^+^ values >5.5 mEq/L (moderate HK or higher; *P* < .05), compared with patients with sK^+^ >5.0–5.5 mEq/L (mild HK) and ≤5.0 mEq/L (normal sK^+^), in whom the monthly change in sK^+^ was minimal and did not differ between groups (Table 2). The greater sK^+^ concentration reductions at higher sK^+^ values were also observed in the piecewise linear mixed model fit of sK^+^, showing the decrease in sK^+^ after initiating patiromer (Fig. 2). In subanalyses by sK^+^ concentration categories (Fig. 3), patiromer decreased sK^+^ to the greatest extent in those with an sK^+^ of >5.5–6.5 mEq/L. Sensitivity analyses extending the linear mixed model to include patients treated with either patiromer or sodium zirconium cyclosi licate yielded results consistent with the primary analysis, confirming robustness across contemporary potassium binders (Supplementary Table S7 and Supplementary Fig. S1).

### RASi/MRA Optimization in Propensity Score-matched Patiromer-treated and Not Treated With Potassium Binder Patients

**Fig 4.**
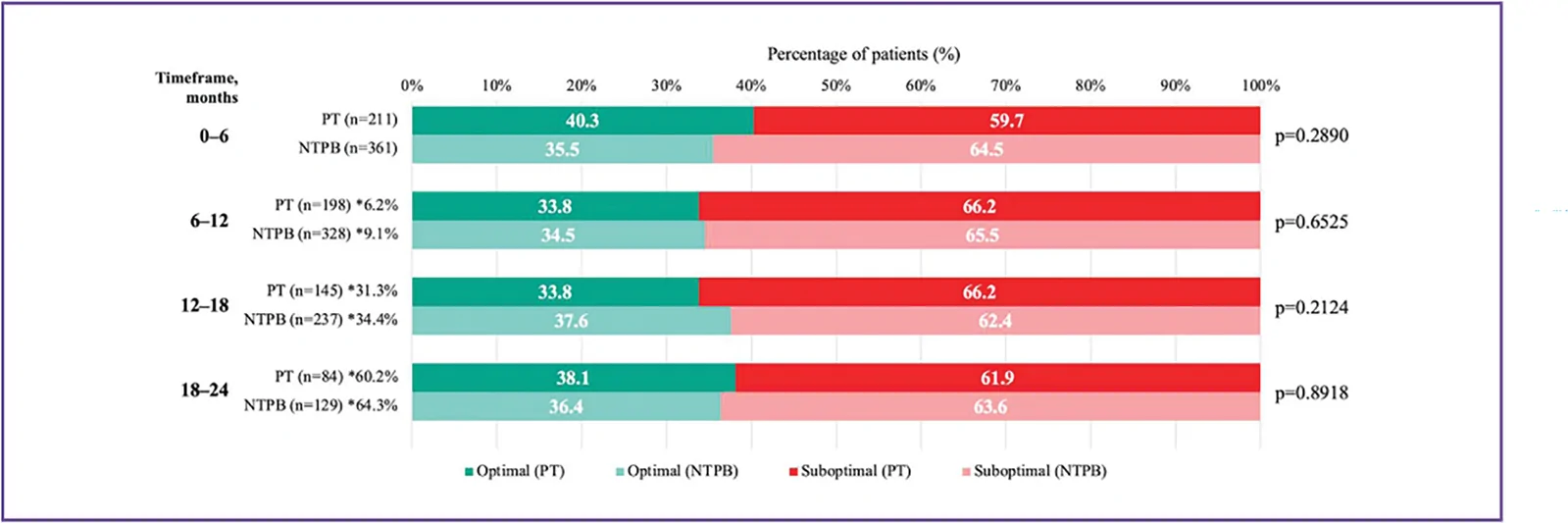
RASi/MRA optimization in each 6-month period in the propensity score-matched patiromer-treated vs not treated with potassium binder patients after a hyperkalemia episode. *Percentages denote missing data at each time interval relative to the baseline-matched cohort (PT *n* = 211; NTPB *n* = 361 at 0–6 months). Optimal: ≥50% of the guideline-recommended dose for ≥1 ACEi/ARB/ARNi, and ≥50% of the guideline-recommended dose for MRA. Suboptimal: <50% of the guideline-recommended dose for all ACEi/ARB/ARNi, or <50% of the guideline-recommended dose for MRA, including untreated patients. For propensity score-matched PT patients, RASi/MRA optimization is observed at 6-month intervals after the last HK episode before patiromer initiation. For propensity score-matched NTPB patients, RASi/MRA optimization is observed at 6-month intervals after the first hyperkalemia episode of the analyzed period. *P* value calculated with conditional logistic regression to compare proportions in matched patients. ACEi, angiotensin-converting enzyme inhibitor; ARB, angiotensin II receptor blocker; ARNi, angiotensin receptor-neprilysin inhibitor; MRA, mineralocorticoid receptor antagonist; NTPB, not treated with potassium binder; PT, patiromer-treated; RASi, renin–angiotensin system inhibitor;.

Optimal RASi/MRA treatment was achieved in 34%–40% of patiromer-treated and not treated with potassium binder patients over the 2 years after an HK episode, with similar rates observed between patiromer-treated patients and patients not treated with a potassium binder across each 6-month period through 2 years (Fig. 4). There was a similar improvement in the best optimization of RASi/MRA achieved in the patiromer-treated patients compared with those not treated with a potassium binder (40.3% vs 35.5%) at the 0- to 6-month time point after an HK episode (odds ratio 0.81; 95% confidence interval [CI] 0.53–1.26). The mixed-effects logistic regression analysis, adjusted for sK^+^ at the time of the HK episode owing to imbalance in this covariate among matched patients revealed an unclear relationship between patiromer use and RASi/MRA optimization across 6-month time points; however, in the first 6 months, where the sample size was largest, patients receiving patiromer had a 19% lower odds of being suboptimally treated or not treated, compared with those in the control group (Supplementary Table S8).

## DISCUSSION

In this contemporary multinational cohort of patients with HF at high risk of HK, <1 in 10 patients were initiated on patiromer upon being deemed at increased risk of HK. Most patients were White men in their early 70s, nearly one-half with CKD stage ≥3b, approximately 7 of 10 patients had HF with reduced ejection fraction, and most had mild to moderate HK. Patiromer initiation resulted in greater sK^+^ reductions, mainly in patients with a baseline sK^+^ of >5.5 to 6.5 mEq/L compared with those with a baseline sK^+^ of ≤ 5.5 mEq/L. However, optimal RASi/MRA treatment was received in <4 in 10 patients over 2 years, with no differences between the patiromer-treated and the nontreatment with potassium binder groups. Notably, 94.4% of HK episodes leading to patiromer initiation did not prompt RASi/MRA modification within the subsequent month.

To our knowledge, this is the first prospective registry to report on the use of potassium binders in the routine clinical practice of patients with HF. The baseline characteristics of the CARE-HK in HF registry were broadly comparable with those of the DIAMOND and PEARL-HF trials.^15,16^ Notable differences included that the registry patients were older and had higher baseline sK^+^.

In CARE HK in HF, patiromer initiation was associated with a larger decrease in sK^+^ when initiated at higher sK^+^ values (>5.5–≤6.5 mEq/L), with the major sK^+^ change of −1.01 mEq/L/month (95% CI −1.86 to −0.16, *P* = .02) observed at sK^+^ concentrations of >6.5 mEq/L. Similar results were observed in the OPAL-HK (A Two-Part, Single-Blind, Phase 3 Study Evaluating the Efficacy and Safety of Patiromer for the Treatment of Hyperkalemia), randomized, placebo-controlled trial which included patients receiving RASi/MRA with CKD and HK, of whom 42% had HF. Patiromer prompted an adjusted mean change ± standard error (adjusted for presence or absence of HF and type 2 diabetes, and baseline sK^+^) from baseline in sK^+^ at 4 weeks of −1.01 ± 0.03 mEq/L (*P* < .001), and 76% of patiromer-treated patients achieved normal sK^+^ values at 4 weeks.^19^ The DIAMOND trial in HF patients with RASi/MRA-associated HK resulted in a mean adjusted sK^+^ difference of −0.10 mEq/L (95% CI −0.13 to 0.07, *P* < .001), and a 37% lower risk of HK with patiromer (hazard ratio 0.63, 95% CI 0.45, 0.87, *P* = .006) when compared with placebo.^16^ However, in our study, patiromer had a minimal effect on sK^+^ concentrations in patients with a normal sK^+^ (≤5.0 mEq/L) or mild HK (>5.0–5.5 mEq/L), suggesting that patients in these ranges are at a lower risk of developing hypokalemia while on patiromer.

In our study, optimal RASi/MRA was received in <4 in 10 patients over 2 years after an HK episode, without differences between patiromer-treated patients and those not treated with potassium binders. Previous studies, some in more controlled settings, have assessed the role potassium binders have in achieving optimal RASi/MRA use.^22–24^ Notably, in the DIAMOND trial, patients had to undergo a run-in phase to be eligible for randomization. In this phase, all patients received patiromer, with approximately 85% of patients with current HK or a history of HK achieving optimal RASi/MRA and an sK^+^ of ≤5.0 mEq/L.^24^ A recent meta-analysis pooling contemporary potassium binder trials data found that potassium binders were associated with successful MRA optimization, although not RASi optimization, compared with placebo.^25^

Recently updated HF guidelines include detailed recommendations on the timing of sK^+^ concentration monitoring when receiving an RASi/MRA, on the management of HK, and on when to start potassium binders to allow for the initiation or uptitration of GDMT.^10,21^ However, despite the close monitoring of sK^+^ recommendations, 55%–93% of patients with HF do not have laboratory follow-up after an HK episode or when starting a RASi/MRA.^19^ This finding is consistent with the extent of missing sK^+^ data during our follow-up study. National HF registries could begin including potassium binders in their data collection forms for future research and undertake quality improvement initiatives to further assess real-world data on optimal GDMT practices at the time of discharge from an HF hospitalization.

Many factors, including CKD, HK, and low blood pressure, are associated with the undertreatment of HF.^1,3,6^ However, a culture of clinical inertia—characterized by minimal tolerance for medication side effects—likely hinders treatment intensification and the achievement of optimal GDMT. This potentially explains our findings of no difference in RASi/MRA optimization despite improved sK^+^ value, and aligns with the CHAMP-HF registry, which indicates that medication adjustments are infrequent during follow-up.^1,3^ Our results highlight how managing sK^+^ concentration alone is not enough to achieve optimal GDMT. Therefore, targeted quality improvement initiatives are needed to overcome clinical inertia and increase the optimal treatment rates of HF, which could subsequently improve outcomes.

## LIMITATIONS

Several limitations of this study should be noted. Although the CARE-HK in HF registry had broad eligibility criteria compared with clinical trials, participation was limited to patients who provided consent, introducing potential selection bias. Although the cohort’s demographics were comparable with those in clinical trials, most participants were from Europe. They were predominantly White men, reflecting the demographic distribution of many participating European countries and limiting generalizability to a broader population. Although approximately 300 patiromer-treated patients were required to power this exploratory analysis adequately, only 211 were included in the propensity score–matched cohort owing to the early termination of the registry, which reflects the real-world underuse of potassium binders. This reduced sample size should be considered when interpreting the results. Despite careful propensity score matching, this observational study cannot establish definitive causation. Perfect balance across all covariates between groups is not guaranteed and subsequent matched analyses were adjusted for the imbalanced covariate, sK^+^ at the time of the HK episode. Moreover, data entry from electronic health records may be subject to errors owing to documentation variability and potential misclassification of diagnoses or medications. Finally, missing sK^+^ data during follow-up was a limitation, consistent with studies reporting inadequate monitoring after HK episodes.

## CONCLUSIONS

Among patients in the CARE-HK in HF registry, only 17% of patients received any potassium binder, and just 9% were prescribed patiromer. Potassium binders were primarily used to treat HK rather than for prophylactic prevention. Patiromer was associated with greater reductions in sK^+^ values at higher baseline sK^+^. However, optimal RASi/MRA treatment was received by only 34%–40% of patients, with little difference between those who were or were not treated with patiromer. Overcoming therapeutic inertia remains problematic despite the availability of effective agents to control HK.

## Supplementary Material

Supplement

Supplementary material associated with this article can be found in the online version at doi:10.1016/j.cardfail.2026.03.025.

## Figures and Tables

**Table 1:** Characteristics of Propensity Score-matched Patiromer-treated and Not Treated With Potassium Binder Groups at the Time of Analysis*

	Overall (*n* = 572)	Patiromer-treated (*n* = 211)	Not Treated With Potassium Binder (*n* = 361)

Age, years, median (IQR)	73 (64–79)	73 (64–78)	73 (64–80)
Age, n (%)*			
<65 years	150 (26.2)	54 (25.6)	96 (26.6)
65–<75 years	177 (30.9)	68 (32.2)	109 (30.2)
≥75 years	245 (42.8)	89 (42.2)	156 (43.2)
Sex, n (%)			
Male	454 (79.4)	166 (78.7)	288 (79.8)
Female	118 (20.6)	45 (21.3)	73 (20.2)
Region, n (%)			
Europe	512 (89.5)	192 (91.0)	320 (88.6)
United States	60 (10.5)	19 (9.0)	41 (11.4)
Race, n (%)^†^			
White	505 (88.3)	186 (88.2)	319 (88.4)
Other	22 (3.8)	8 (3.8)	14 (3.9)
Unknown or not available	45 (7.9)	17 (8.1)	28 (7.8)
No. of comorbidities, n (%)^*,†^			
≤1	71 (12.4)	26 (12.3)	45 (12.5)
2	41 (7.2)	15 (7.1)	26 (7.2)
3	77 (13.5)	26 (12.3)	51 (14.1)
>3	383 (67.0)	144 (68.2)	239 (66.2)
Diabetes, n (%)*			
Yes	287 (50.2)	107 (50.7)	180 (49.9)
No	285 (49.8)	104 (49.3)	181 (50.1)
CKD stage, n (%)^*,†^			
Stage 1–3a	260 (45.5)	91 (43.1)	169 (46.8)
Stage 3b–5	257 (44.9)	101 (47.9)	156 (43.2)
Missing	55 (9.6)	19 (9.0)	36 (10.0)
Most recent LVEF at enrollment, n (%)^†^			
HFrEF	407 (71.2)	153 (72.5)	254 (70.4)
HFmrEF	98 (17.1)	36 (17.1)	62 (17.2)
HFpEF	65 (11.4)	21 (10.0)	44 (12.2)
Missing	2	1	1
NYHAfunctional classification			
I	102 (17.8)	41 (19.4)	61 (16.9)
II	345 (60.3)	125 (59.2)	220 (60.9)
III	97 (17.0)	35 (16.6)	62 (17.2)
IV	9 (1.6)	3 (1.4)	6 (1.7)
Missing	19	7	12
sK^+^ (mEq/L) at analysis, median (IQR)^‡,§^	5.5 (5.3, 5.7)	5.5 (5.4, 5.7)	5.4 (5.2, 5.6)
Available follow-up time (months), median (IQR)	13.3 (9.1,20.2)	12.3 (7.7, 20.6)	13.8 (9.5,19.9)

* Analysis date in patiromer-treated patients = at the time of the last HK episode before or at the time of patiromer initiation; not treated with potassium binder = at the time of the first HK episode reported in the registry period.

† Recategorization was required in some instances because the number of patients in each category was too low for matching; therefore, categories were combined.

‡ For sK^+^, log-transformed variables were used in the matching process.

§ sK^+^ at the time of the hyperkalemia episode was still not balanced after matching and included in the propensity score analysis. For patients with no sK^+^ available before analysis date, sK^+^ was imputed to the median of values in patients with available value in the patiromer-treated and not treated with potassium binder, respectively.

All other characteristics analyzed for propensity score matching (not shown) had broadly similar data between the two populations.

CKD, chronic kidney disease; HFmrEF, heart failure with mildly reduced ejection fraction; HFpEF, heart failure with preserved ejection fraction; HFrEF, heart failure with reduced ejection fraction; IQR, interquartile range; LVEF, left ventricular ejection fraction; NYHA, New York Heart Association; sK^+^, serum potassium.

**Table 2:** Change in sK^+^ Before and After Patiromer Initiation of All Patiromer-treated Patients (*n* = 234)

	Estimated Slope Difference (mEq/L/month)	95% CI	*P* value

Overall	−0.21	(−0.30 to −0.12)	**<.0001**
By sK^+^ value category at time of patiromer initiation			
≤5.0 mEq/L	−0.04	(−0.31 to 0.24)	.79
>5.0−5.5 mEq/L	−0.07	(−0.24 to 0.10)	.41
>5.5−6.0 mEq/L	−0.17	(−0.32 to −0.02)	**.02**
>6.0−6.5 mEq/L	−0.48	(−0.67 to −0.29)	**<.0001**
>6.5 mEq/L	− 1.01	(−1.86 to −0.16)	**.02**

Estimates from a linear mixed model with sK^+^ values as the dependent variable, time (continuous, in months, calculated as the difference between sK^+^ measurement date and patiromer initiation date divided by 30.4), time spline (equal to 0 before patiromer initiation, and equal to time after patiromer initiation) as fixed effects and patient as random effects. For analysis by subgroup, the subgroup variable (categorical) and its interaction with time and time spline were added as fixed effects. Patiromer initiation date is used as a knot. The analysis produced a slope estimate of the sK^+^ values before and after patiromer initiation separately. This table presents the change in these two slopes (before−after), with a negative change demonstrating an overall decrease in sK^+^ after patiromer initiation.

sK^+^ values measured before 3 months or after 6 months from patiromer initiation were excluded from the analysis. Values below 2 mEq/L and above 9 mEq/L were considered to be clinically invalid and were excluded from the analysis. Duplicated values of sK^+^ reported on the same day for the same patient were excluded from analysis, only distinct values were considered in the model.

CI, confidence interval; sK^+^, serum potassium.
