# Supplementary material for: Patiromer for Patients With Heart Failure at Risk of Hyperkalemia: CARE-HK in HF Registry
Source: J Card Fail. Author manuscript; Available in PMC 2026 Sep 21. (PMC13592558; doi:10.1016/j.cardfail.2026.03.025)
Supplement: Supplementary file 1 [file NIHMS2208278-supplement-Supplement.docx]

**Supplemental Material**

**Supplemental Fig. 1.** Piecewise linear mixed model of serum potassium values before and after patiromer or sodium zirconium cyclosilicate initiation (n=335).

sK+, serum potassium. SZC: sodium zirconium cyclosilicate.

Linear mixed model with sK+ values as the dependent variable, time (continuous, in months, calculated as the difference between sK+ measurement date and patiromer initiation date divided by 30.4), time spline (equal to 0 prior to patiromer initiation, and equal to time after patiromer initiation) as fixed effects and patient as random effects. Patiromer initiation date is used as a knot.

The blue line indicates the regression line. The red dashed line indicates the serum potassium concentration threshold for hyperkalemia at 5 mEq/L. The circles in the figures illustrate all sK+ readings; all results for a patient are included, so there are multiple sK+ readings displayed.

sK+ values measured prior to 3 months or after 6 months from patiromer initiation were excluded from the analysis. Values below 2 mEq/L and above 9 mEq/L were considered to be clinically invalid and are excluded in the analysis. Duplicated values of sK+ reported on the same day for the same patient were excluded from analysis, only distinct values were considered in the models.

**Supplemental Table 1.** Covariate balance assessment before and after propensity score matching

|  | **SMD** | | **VR** | |
| --- | --- | --- | --- | --- |
|  | **Before PS matched n = 1386** | **After PS matched  n = 572** | **Before PS matched n = 1386** | **After PS matched  n = 572** |
| **Age category at HK episode, years** |  |  |  |  |
| <65 | –0.0145 | –0.0228 | 0.9841 | 0.9755 |
| ≥65 to <75 | –0.0303 | 0.0433 | 0.9783 | 1.0363 |
| ≥75 | 0.0419 | –0.0210 | 1.0152 | 0.9939 |
| **Sex** | 0.1735 | –0.0256 | 0.8179 | 1.0401 |
| **Country** |  |  |  |  |
| Austria | 0.1340 | 0.0961 | 1.3970 | 1.2512 |
| Belgium | 0.1809 | 0.0500 | 1.9121 | 1.1453 |
| Switzerland | 0.0507 | 0.0009 | 1.1801 | 1.0027 |
| Germany, UK | –0.4077 | –0.0461 | 0.1751 | 0.6295 |
| Spain | 0.2761 | –0.0502 | 1.3076 | 0.9724 |
| Italy | 0.3100 | 0.0381 | 1.6946 | 1.0441 |
| USA | –0.6155 | –0.0605 | 0.3708 | 0.8139 |
| Race |  |  |  |  |
| White | 0.1629 | –0.0061 | 0.7201 | 1.0159 |
| Other | –0.1041 | –0.0040 | 0.6428 | 0.9785 |
| Unknown | –0.1180 | 0.0101 | 0.7245 | 1.0354 |
| **Reason for RASi/MRA recommendation at enrollment** |  |  |  |  |
| HFrEF | 0.2121 | 0.0775 | 0.7408 | 0.8772 |
| HFmrEF | 0.0195 | –0.0492 | 1.0659 | 0.8675 |
| HFpEF | –0.2592 | –0.0547 | 0.5720 | 0.8523 |
| **Time from RASi/MRA initiation to HK episode, years** | 0.0666 | –0.0010 | 1.0820 | 1.0567 |
| **Diabetes at HK episode** | 0.0964 | 0.0170 | 1.0066 | 0.9998 |
| **Resistant hypertension at HK episode** | 0.0221 | 0.0474 | 1.1264 | 1.3194 |
| **Number of comorbidities at HK episode** |  |  |  |  |
| 0 or 1 | 0.0235 | –0.0044 | 1.0566 | 0.9901 |
| 2 | –0.0686 | –0.0034 | 0.8085 | 0.9880 |
| 3 | 0.0127 | –0.0553 | 1.0300 | 0.8906 |
| >3 | 0.0147 | 0.0437 | 0.9888 | 0.9686 |
| **sK+ value at HK episode (imputed),  log mEq/L** | 0.5447 | **0.1840** | 0.6184 | 0.5952 |
| **NT-proBNP after HK episode (imputed)** | 0.2396 | 0.0584 | 0.9698 | 0.9797 |
| **CKD stage at HK episode** |  |  |  |  |
| 1 | –0.0466 | –0.0259 | 0.8373 | 0.9019 |
| 2 | –0.2157 | –0.0334 | 0.7255 | 0.9383 |
| 3a | –0.0111 | –0.0421 | 0.9851 | 0.9470 |
| 3b | 0.1021 | 0.0753 | 1.1111 | 1.0774 |
| 4 | 0.1028 | 0.0557 | 1.2153 | 1.1033 |
| 5 | –0.0475 | –0.0654 | 0.6496 | 0.5744 |
| Missing | 0.0915 | –0.0362 | 1.3373 | 0.9127 |
| **LVEF at enrollment** |  |  |  |  |
| HFrEF | 0.2229 | 0.0462 | 0.8471 | 0.9558 |
| HFmrEF | 0.0249 | –0.0030 | 1.0458 | 0.9948 |
| HFpEF | –0.3251 | –0.0621 | 0.5282 | 0.8374 |
| **NYHA classification** |  |  |  |  |
| I | 0.0840 | 0.0663 | 1.1520 | 1.1149 |
| II | 0.1943 | –0.0343 | 0.9659 | 1.0144 |
| III | –0.0471 | –0.0155 | 0.9224 | 0.9727 |
| IV | –0.0309 | 0.0191 | 0.7878 | 0.8575 |
| Missing | –0.3863 | –0.0002 | 0.2668 | 0.9981 |
| **Available follow-up time, months** | –0.3868 | 0.0151 | 0.8296 | 1.2612 |
| **SGLT2 inhibitor use at HK episode** | 0.3333 | 0.0023 | 0.9548 | 0.9989 |
| **ARNI use at HK episode** | 0.1303 | –0.0317 | 0.9812 | 1.0099 |

An improvement in the covariates balance after matching would be indicated by smaller absolute values of SMDs and variance ratios closer to 1 after matching. A negative SMD indicates a higher value of the covariate or frequency of the subcategory in the non-potassium binder group.
For the patiromer treated group, variables were assessed at the time of the last HK episode prior to or at the time of patiromer initiation. For the not treated with potassium binder group, variables were assessed at the time of the first HK episode reported in the registry period.
Some categories were combined due to low numbers of patients on patiromer.
CKD, chronic kidney disease; HfmrEF, heart failure with midrange ejection fraction; HfpEF, heart failure with preserved ejection fraction; HfrEF, heart failure with reduced ejection fraction; HK, hyperkalemia; LVEF, left ventricular ejection fraction; MRA, mineralocorticoid receptor antagonist; NT-proBNP, N-terminal pro-B-type natriuretic peptide; NYHA, New York Heart Association; PS matched, patients matched using propensity score model; RASi, renin–angiotensin system inhibitor; sK+, serum potassium; SMD, standardized mean difference; VR, variance ratio.

**Supplemental Table 2.** Potassium binder therapies used

|  | **Overall**  **(N = 2558)** | **United States**  **(N = 832)** | **Europe**  **(N = 1726)** | ***P*-value** |
| --- | --- | --- | --- | --- |
| **Number of patients on potassium binders, n (%)** | 434 (17.0) | 55 (6.6) | 379 (22.0) |  |
| **Patiromer^a^** | 234 (9.1) | 22 (2.6) | 212 (12.3) | <0.001 |
| *Reason for use* |  |  |  |  |
| To treat hyperkalemia^b^ | 199 (85.0) | 21 (95.5) | 178 (84.0) |  |
| To maintain normokalemia^b^ | 43 (18.4) | 3 (13.6) | 40 (18.9) |  |
| To up-titrate or initiate RASi/MRA therapy^b^ | 4 (1.7) | 0 | 4 (1.9) |  |
| Other^b^ | 9 (3.8) | 0 | 9 (4.2) |  |
| **Sodium zirconium cyclosilicate^a,c^** | 150 (5.9) | 21 (2.5) | 129 (7.5) | <0.001 |
| *Reason for use* |  |  |  |  |
| To treat hyperkalemia^b^ | 119 (79.3) | 19 (90.5) | 100 (77.5) |  |
| To maintain normokalemia^b^ | 29 (19.3) | 3 (14.3) | 26 (20.2) |  |
| To up-titrate or initiate RASi/MRA therapy^b^ | 6 (4.0) | 0 | 6 (4.7) |  |
| Other^b^ | 4 (2.7) | 0 | 4 (3.1) |  |
| **Calcium polystyrene sulfonate^a,c^** | 62 (2.4) | 0 | 62 (3.6) | <0.001 |
| *Reason for use* |  |  |  |  |
| To treat hyperkalemia^b^ | 55 (88.7) | 0 | 55 (88.7) |  |
| To maintain normokalemia^b^ | 5 (8.1) | 0 | 5 (8.1) |  |
| To up-titrate or initiate RASi/MRA therapy^b^ | 1 (1.6) | 0 | 1 (1.6) |  |
| Other^b^ | 0 | 0 | 0 |  |
| **Sodium polystyrene sulfonate^a,c^** | 48 (1.9) | 14 (1.7) | 34 (2.0) | 0.61 |
| *Reason for use* |  |  |  |  |
| To treat hyperkalemia^b^ | 45 (93.8) | 13 (92.9) | 32 (94.1) |  |
| To maintain normokalemia^b^ | 5 (10.4) | 2 (14.3) | 3 (8.8) |  |
| To up-titrate or initiate RASi/MRA therapy^b^ | 0 | 0 | 0 |  |
| Other^b^ | 1 (2.1) | 0 | 1 (2.9) |  |

Data reflect n (%). ^a^Percentage calculated among all patients included in the analysis. ^b^Percentage calculated among patients prescribed the specific potassium-controlling agent. ^c^A patient may be classified under more than one potassium binder category.

MRA, mineralocorticoid receptor antagonist; RASi, renin–angiotensin system inhibitor.

This table lists the most commonly used potassium binders but is not an exhaustive list. Patients in the NTPB group were excluded if they received any potassium binder.

**Supplemental Table 3.** Characteristics of all patiromer-treated and not treated with potassium binder groups at time of analysis before propensity score matching.^a^

|  | Patiromer-treated  (n = 234) | Not treated with potassium binder  (n = 1169) |
| --- | --- | --- |
| Age in years, median (IQR) | 73 (64, 78) | 72 (64, 79) |
| Age, n (%)^a^ |  |  |
| <65 years | 61 (26.1) | 304 (26.2) |
| 65–<75 years | 73 (31.2) | 390 (33.6) |
| ≥75 years | 100 (42.7) | 465 (40.1) |
| Sex, n (%) |  |  |
| Male | 185 (79.1) | 834 (71.3) |
| Female | 49 (20.9) | 335 (28.7) |
| Region, n (%) |  |  |
| Europe | 212 (90.6) | 786 (67.2) |
| US | 22 (9.4) | 383 (32.8) |
| Race, n (%)^b^ |  |  |
| White | 207 (88.5) | 965 (82.5) |
| Other | 8 (3.4) | 70 (6.0) |
| Unknown or Not Available | 19 (8.1) | 134 (11.5) |
| Number of comorbidities, n (%)^a,b^ |  |  |
| ≤1 | 29 (12.4) | 144 (12.3) |
| 2 | 16 (6.8) | 104 (8.9) |
| 3 | 30 (12.8) | 138 (11.8) |
| >3 | 159 (67.9) | 783 (67.0) |
| Diabetes, n (%)^a^ |  |  |
| Yes | 116 (49.6) | 532 (45.5) |
| No | 118 (50.4) | 637 (54.5) |
| CKD stage, n (%)^a,b^ |  |  |
| Stage 1 to 3a | 94 (40.2) | 618 (52.9) |
| Stage 3b to 5 | 109 (46.6) | 465 (39.8) |
| Missing | 31 (13.2) | 86 (7.4) |
| Most recent LVEF at enrollment, n (%)^b^ |  |  |
| HFrEF | 163 (70.0) | 727 (62.2) |
| HFmrEF and HFpEF | 70 (30.0) | 441 (37.8) |
| Missing | 1 | 1 |
| sK^+^ (mEq/L) at analysis date, median (IQR)^c,d^ | 5.5 (5.4, 5.6) | 5.3 (5.2, 5.5) |
| Available follow-up time (months), median (IQR) | 13.4 (8.9, 24.4) | 18.1 (12.2, 24.7) |
| ARNI at analysis date, n (%) | 122 (52.1) | 567 (48.5) |
| SGLT2 inhibitor at analysis date, n (%) | 121 (51.7) | 469 (40.1) |

^a^Analysis date in patiromer-treated = the time of the last HK episode prior to or at the time of patiromer initiation; not treated with potassium binder = the time of the first HK episode reported in the registry period.

^b^Recategorization was required in some instances because the number of patients in each category was too low for matching; therefore, categories were combined.

^c^For sK^+^, log-transformed variables were used in the matching process.

^d^sK^+^ at the time of the hyperkalemia episode was still not balanced after matching and included in the propensity score analysis. For patients with no sK^+^ available prior to analysis date, sK^+^ was imputed to the median of values in patients with available value in the patiromer-treated and not treated with potassium binder, respectively.

All other characteristics analyzed for propensity score matching (not shown) had broadly similar data between the two populations.

ARNI, Angiotensin Receptor-Neprilysin Inhibitor; CKD, chronic kidney disease; HFmrEF, heart failure with mildly reduced ejection fraction; HFpEF, heart failure with preserved ejection fraction; HFrEF, heart failure with reduced ejection fraction; IQR, interquartile range; LVEF, left ventricular ejection fraction; SGLT2, Sodium-Glucose Cotransporter 2; sK+, serum potassium; US, United States.

**Supplemental Table 4.** RASi/MRA treatment modifications following the last HK episode prior to patiromer initiation in RASi-treated patients.

| Action | At short term (30 days)^a^  n = 72^b^ |
| --- | --- |
| No RASi/MRA modification, n (%)^c^ | 68 (94.4) |
| Down-titration, n (%)^c^ | 1 (1.4) |
| Up-titration, n (%)^d^ | 0 |
| Up-titration to RASi/MRA optimization, n (%)^d^ | 0 |
| Interruption, n (%)^d^ | 1 (1.4) |
| Reinitiation, n (%)^e^ | 0 |
| Reinitiation to RASi/MRA optimization, n (%)^e^ | 0 |
| Discontinuation, n (%) | 2 (2.8) |

^a^Short term: this period extends up to 30 days after the HK episode, or up to the day before the start date of a recurrent HK episode, or up to the follow-up end date, whichever occurs first.

^b^Limited to patients with a HK episode within 3 months prior to or at patiromer initiation; earlier HK episodes (>3 months prior) were excluded as they were unlikely to trigger patiromer therapy.

^c^Percentage among all patients analyzed for the period.

^d^Percentage among patients with a down-titration.

^e^Percentage among patients with an interruption.

HK, hyperkalemia; MRA, mineralocorticoid receptor antagonist; RASi, renin–angiotensin system inhibitor.

**Supplemental Table 5.** Patiromer treatment duration and patient disposition in patiromer-treated, not treated with potassium binder, and propensity score-matched populations overall and by region

|  | Overall | Europe | US |
| --- | --- | --- | --- |
| CARE HK registry cohort, n^a^ | 2558 | 1726 | 832 |
| Patiromer-treated^b^, n (%) | 234 (9.1) | 212 (12.3) | 22 (2.6) |
| Total duration of patiromer treatment in months^c^, median (IQR) | 12.0 (6.0–19.4) | 12.0 (6.0–19.2) | 8.8 (3.9–21.5) |
| Discontinued patiromer^d^, n (%) | 136 (58.1%) | 117 (55.2%) | 19 (86.4%) |
| PS-matched patiromer-treated, n | 211 | 192 | 19 |
| Total duration of patiromer treatment in  PS-matched patients in months, median (IQR) | 11.4 (6.0–16.3) | 11.7 (6.0–16.2) | 7.4 (3.9–20.6) |
| Not treated with potassium binder^e^, n (%) | 1169 (45.7) | 786 (45.5) | 383 (46.0) |
| PS-matched not treated with potassium binder, n | 361 | 320 | 41 |

^a^Included patients in the CARE HK registry (full analysis set).

^b^Patients from the total population who received patiromer prior to enrollment or during follow-up. Percentage calculated among all patients included in the analysis.

^c^Total duration of patiromer treatment: calculated as the sum of all treatment periods.

^d^Patients who discontinued patiromer over the whole study period, with percentages calculated from all patiromer-treated patients.

^e^Patients from the total population who did not receive any potassium binder prior to enrollment or during follow-up, with ≥1 hyperkalemia episode over the whole study period. Percentage among the total population.

Documented reasons for patiromer discontinuation included maintenance of normal serum potassium levels (n=51, 50.5%), RASi/MRA uptitration (n=5, 5.0%), concerns regarding effectiveness (n=5, 5.0%), concerns for hypokalemia (n=8, 7.9%), patient decision to decline continued patiromer therapy (n=13, 12.9%), lack of insurance coverage (n=1, 1.0%), and other reasons (n=39, 38.6%); the reason for discontinuation was missing in 35 patients.

Among the PS-matched patiromer-treated cohort, patiromer was discontinued in 123 of 211 patients (58.3%). Documented reasons for discontinuation included maintenance of normal serum potassium levels (n=44, 49.4%), RASi/MRA uptitration (n=5, 5.6%), concerns regarding effectiveness (n=4, 4.5%), concerns for hypokalemia (n=5, 5.6%), patient decision to decline continued patiromer therapy (n=13, 14.6%), and other reasons (n=36, 40.4%); the reason for discontinuation was missing in 34 patients.

IQR, interquartile range; PS, propensity score; US, United States.

**Supplemental Table 6.** Serum potassium values before and after patiromer initiation in the patiromer-treated group before propensity score-matching

| **Timepoint** | **sK^+^ value (mEq/L)** |
| --- | --- |
| 6 months prior to patiromer initiation (n = 46, missing n = 188), median (IQR) | 5.1 (4.7, 5.5) |
| 3 months prior to patiromer initiation (n = 70, missing n = 164), median (IQR) | 5.3 (4.9, 5.6) |
| At patiromer initiation (n = 198, missing n = 36), median (IQR) | 5.6 (5.3, 5.8) |
| 1 month after patiromer initiation (n = 66, missing n = 168), median (IQR) | 5.0 (4.6, 5.4) |
| 3 months after patiromer initiation (n = 108, missing n = 126), median (IQR) | 5.0 (4.6, 5.3) |
| 6 months after patiromer initiation (n = 102, missing n = 132), median (IQR) | 5.0 (4.6, 5.2) |

The closest sK^+^ value assessed within the window before or after the time point is described. Number missing is from the total 234 patiromer-treated patients.

IQR, interquartile range; sK+, serum potassium.

**Supplemental Table 7.** Change in sK+ before/after patiromer or sodium zirconium cyclosilicate initiation of all patiromer or sodium zirconium cyclosilicate treated patients^a^ (n=335).

|  | Estimated slope difference  (mEq/L/month) | 95% CI | *P*-value |
| --- | --- | --- | --- |
| Overall | –0.19 | (–0.26; –0.11) | **<0.0001** |
| By sK+ value category at time of patiromer initiation |  |  |  |
| ≤5.0 mEq/L | –0.05 | (–0.33; 0.23) | 0.72 |
| >5.0–5.5 mEq/L | –0.07 | (–0.24; 0.10) | 0.40 |
| >5.5–6.0 mEq/L | –0.16 | (–0.31; –0.01) | **0.03** |
| >6.0–6.5 mEq/L | –0.53 | (–0.72; –0.33) | **<0.0001** |
| >6.5 mEq/L | –1.01 | (–1.88; –0.14) | **0.02** |

SZC: sodium zirconium cyclosilicate.

^a^The total of 335 patients treated with either patiromer or sodium zirconium cyclosilicate is not simply the sum of 234 patiromer-treated and 150 sodium zirconium cyclosilicate -treated patients, as some received both therapies and populations overlapped. Of the 366 patients treated with either drug, 335 were included in this analysis after excluding those with erroneous sK⁺ values (see below).

Estimates from a linear mixed model with sK+ values as the dependent variable, time (continuous, in months, calculated as the difference between sK+ measurement date and patiromer initiation date divided by 30.4), time spline (equal to 0 prior to patiromer initiation, and equal to time after patiromer initiation) as fixed effects and patient as random effects. For analysis by subgroup, the subgroup variable (categorical) and its interaction with time and time spline were added as fixed effects. Patiromer initiation date is used as a knot. The analysis produced a slope estimate of the sK+ values before and after patiromer initiation separately. This table presents the change in these two slopes (before–after), with a negative change demonstrating an overall decrease in sK+ after patiromer initiation.

CI, confidence interval; sK+, serum potassium.

sK+ values measured prior to 3 months or after 6 months from patiromer initiation were excluded from the analysis. Values below 2 mEq/L and above 9 mEq/L were considered to be clinically invalid and were excluded in the analysis. Duplicated values of sK+ reported on the same day for the same patient were excluded from analysis, only disctinct values were considered in the model.

**Supplemental Table 8.** Mixed effects logistic regression assessing the association between best RASi/MRA optimization at 6-month intervals following a hyperkalemia episode in propensity score-matched patiromer-treated patients.

| Time point | Category | Suboptimal/not treated vs Optimal  RASi/MRA treatment  OR (95% CI) | *P*-value |
| --- | --- | --- | --- |
| 0‒6 months | NTPB (n = 361) | Reference |  |
|  | PT (n = 211) | 0.81 (0.53, 1.26) | 0.35 |
| 6‒12 months | NTPB (n = 328) | Reference |  |
|  | PT (n = 198) | 1.02 (0.64, 1.61) | 0.95 |
| 12‒18 months | NTPB (n = 237) | Reference |  |
|  | PT (n = 145) | 1.20 (0.71, 2.03) | 0.49 |
| 18‒24 months | NTPB (n = 129) | Reference |  |
|  | PT (n = 84) | 0.93 (0.48, 1.81) | 0.82 |

Covariates are assessed at the time of the last HK episode prior to or at the time of patiromer initiation in the PT group and at the time of the first document HK episode reported in the registry period for the NTPB group.

The probability modeled is the probability of having suboptimal RASi/MRA optimization. The treatment group was added as a fixed effect, and random intercept terms were added for patient and matched set. In addition, 6-month timepoints and sK+ were added as covariates (since sK+ remained unbalanced following propensity score matching). Changes in treatment effect over 6-month time points explored by including treatment by visit interactions. OR compare PT to NTPB. NTPB is the reference group. OR <1 suggest patiromer is associated with lower odds of suboptimal RASi/MRA optimization.

CI, confidence interval; HK, hyperkalemia; MRA, mineralocorticoid receptor antagonist; NTPB, not treated with potassium binder; OR, odds ratio; PT, patiromer treated; RASi, renin–angiotensin system inhibitor.
